# The Histone Deacetylase Inhibitor I1 Induces Differentiation of Acute Leukemia Cells With MLL Gene Rearrangements *via* Epigenetic Modification

**DOI:** 10.3389/fphar.2022.876076

**Published:** 2022-04-27

**Authors:** Jingfang Yao, Gentao Li, Zihui Cui, Peilei Chen, Jinhong Wang, Zhenbo Hu, Lei Zhang, Liuya Wei

**Affiliations:** ^1^ Laboratory for Stem Cell and Regenerative Medicine, Affiliated Hospital of Weifang Medical University, Weifang, China; ^2^ School of Pharmacy, Weifang Medical University, Weifang, China

**Keywords:** acute leukemia, mixed lineage leukemia rearranged, differentiation therapy, HDAC inhibitor, epigenetic modification

## Abstract

Acute leukemia (AL) is characterized by excessive proliferation and impaired differentiation of leukemic cells. AL includes acute myeloid leukemia (AML) and acute lymphoblastic leukemia (ALL). Previous studies have demonstrated that about 10% of AML and 22% of ALL are mixed lineage leukemia gene rearrangements (MLLr) leukemia. The prognosis of MLLr leukemia is poor and new therapeutics are urgently needed. Differentiation therapy with all-trans-retinoic acid (ATRA) has prolonged the 5-years disease-free survival rate in acute promyelocytic leukemia (APL), a subtype of AML. However, the differentiation therapy has not been effective in other acute leukemia. Here, we aim to explore the cell differentiation effect of the potent HDACs inhibitor, I1, and the possible mechanism on the MLLr-AML and MLLr-ALL cells (MOLM-13, THP-1, MV4-11 and SEM). It is shown that I1 can significantly inhibit the proliferation and the colony-forming ability of MOLM-13, THP-1, MV4-11 and SEM cells by promoting cell differentiation coupled with cell cycle block at G0/G1 phase. We show that the anti-proliferative effect of I1 attributed to cell differentiation is most likely associated with the HDAC inhibition activity, as assessed by the acetylation of histone H3 and H4, which may dictates the activation of hematopoietic cell lineage pathway in both MOLM-13 and THP-1 cell lines. Moreover, the activity of HDAC inhibition of I1 is stronger than that of SAHA in MOLM-13 and THP-1 cells. Our findings suggest that I1, as a chromatin-remodeling agent, could be a potent epigenetic drug to overcome differentiation block in MLLr-AL patients and would be promising for the treatment of AL.

## Introduction

Acute leukemia (AL) includes acute myeloid leukemia (AML) and acute lymphoblastic leukemia (ALL). AML is a hematological malignancy characterized by increased self-renewal of leukemia stem/progenitor cells that are blocked in myeloid differentiation (Yu et al., 2016). ALL represents 25% of all the cancers diagnosed among children younger than 15 years and 0.1% of all the adult cancers (Allen and Ahmed, 2016). Most cases of ALL arise from the immature hematopoietic stem/progenitor cells with self-renewal and differentiation capacity (George et al., 2001). Hence, the blockade of cellular differentiation represents a hallmark of both AML and ALL.

Mixed lineage leukemia (MLL) gene rearrangements (MLLr) were found in about 10% of all the AML cases (Schoch et al., 2003) and in 22% of all the ALL cases (Gole and Wiesmüller, 2015). Despite great improvements in the treatment for ALL, MLLr-ALL have a particularly poor outcome with low survival rate compared with those of other forms of ALL (Wuchter et al., 2000). Similarly, very few MLLr- AML have either a good or an intermediate outcome (Szczepański et al., 2010). Therefore, there is an urgent need for development of new therapeutics for the MLLr-AL. Acute promyelocytic leukemia (APL) is the M3 subtype of AML, which is one of the most aggressive types and accounts for 10–15% of AML (Prange et al., 2017). Fortunately, the differentiation inducer ATRA is effective in the treatment of APL (Wang and Chen, 2008). However, the differentiation therapy with ATRA has not been effective in the treatment of the other subtypes of AML and ALL.

Histone deacetylases (HDACs) are enzymes that remove acetyl groups from histones and other proteins and thus regulate chromatin accessibility and expression of target genes (Manzotti et al., 2019). It is well documented that inhibition of HDACs promotes growth arrest and cell differentiation or apoptosis through altering the acetylation status of histone and non-histone proteins (Rosato et al., 2003; Manal et al., 2016). So far, there are four FDA-approved anticancer drugs targeting HDAC, i.e., SAHA (Suberoylanilide hydroxamic acid), FK-228, PXD-101 and LBH-589 (O'Connor et al., 2006; West and Johnstone, 2014). SAHA is a well-studied and most famous HDAC inhibitor (HDACi), which is used for the treatment of cutaneous T cell lymphoma.

In our previous study, I1 (4-(4-(1H-indol-3-yl)butanamido)-N-hydroxybenzamide, C_19_H_20_N_3_O_3_) is a HDACs inhibitor, which has considerable HDAC inhibitory potency with the percentage inhibitory rate of 53.81% compared with SAHA (59.91%) at the concentration of 1 μM (Chen et al., 2021). In the present study, we evaluate whether I1 has inhibitory activity by inducing cell differentiation on MLLr-AML and MLLr-ALL cell lines (MOLM-13, THP-1, MV4-11 and SEM). The possible mechanism of action of I1 is also explored.

## Materials and Methods

### Chemicals

**FIGURE 1:**
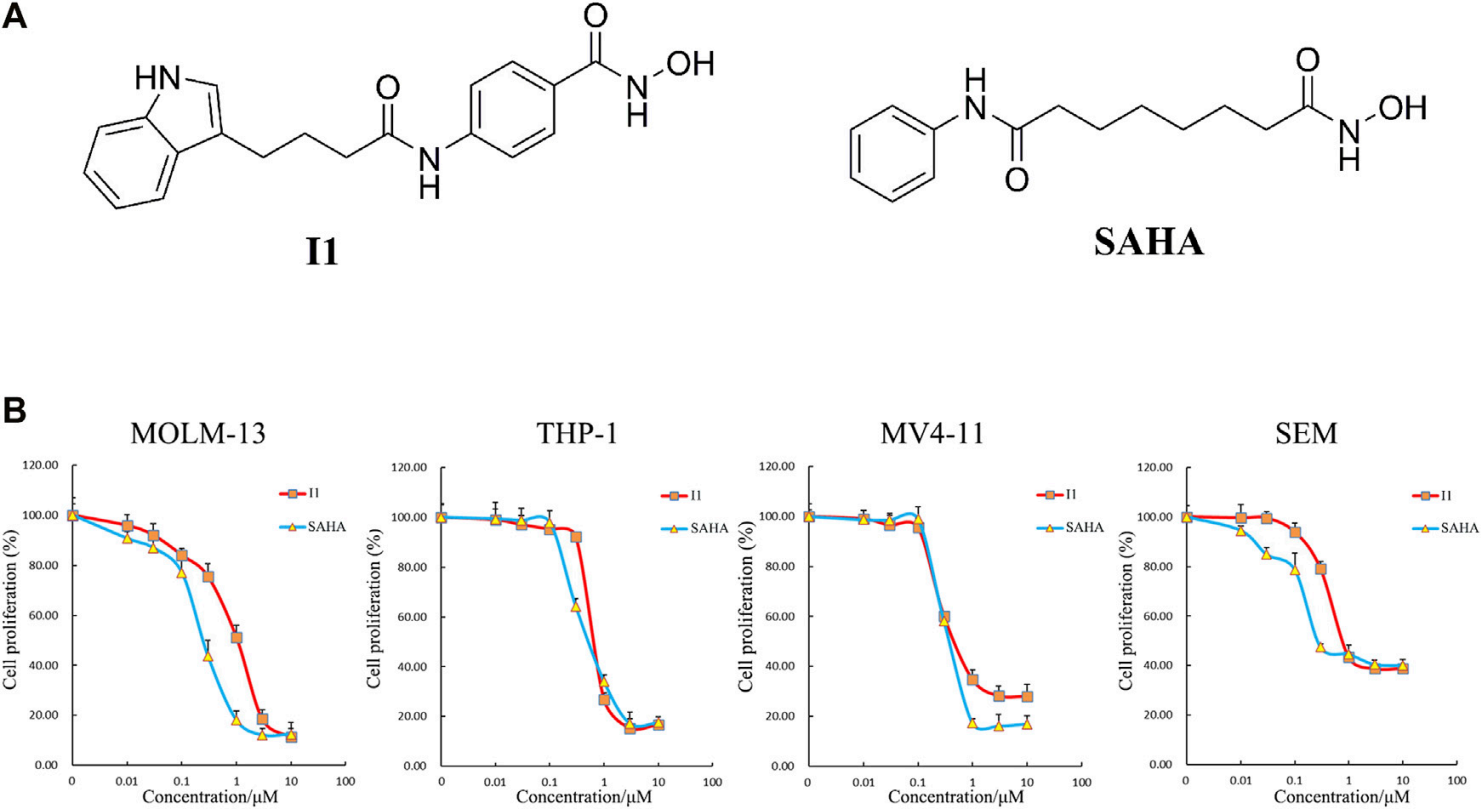
I1 inhibited the proliferation of MOLM-13, THP-1, MV4-11 and SEM cells. **(A)** The chemical structure of I1 and SAHA. **(B)** CCK-8 assay on cells treated with different concentrations of I1 or SAHA (0–10 μM) for 72 h. Data presented as mean ± SD. The CCK-8 assay was carried out for three times in triplicate.

I1 was prepared by our lab. Figure 1A shows the chemical structure of I1 with a molecular weight of 338 and SAHA. I1 or SAHA was dissolved in dimethyl sulfoxide (DMSO) to prepare a 10 mM stock solution and stored at −20°C. The stock solutions were diluted to the desired concentrations with RPMI-1640 medium. The same concentration of DMSO as that of the I1 solution was used as control. The final concentration of DMSO (not exceed 0.1%) in the cell culture had few toxic effect on the cells. Fluorescein Isothiocyanate (FITC)/Annexin V Apoptosis Detection Kit and Propidium iodide (PI)/RNase staining solution were obtained from BD Biosciences (San Jose, CA, United States). Cell Counting Kit-8 (CCK-8) was purchased from Solarbio (Beijing, China). FITC anti- CD11b (cat #301403, RRID:AB_314167), PE anti- CD13 (cat #301704, RRID:AB_314180), FITC anti- CD14 (cat #301804, RRID: AB_314186) and PE anti- CD15 (cat #301906, RRID: AB_314198) were obtained from Biolegend Inc. (San Diego, CA, United States). PE anti-human HLA-DR (FAB4869P) were obtained from R&D systems (Minneapolis, MN, United States). Anti -human HLA-DP (sc-33719) was obtained from Santa Cruz Biotechnology, Inc. (Dallas, TX, United States). MethoCult H4100 (cat #04100) and H4435 (cat #04435) were obtained from STEMCELL Technologies (Vancouver, BC, Canada). Antibodies against GAPDH (Cat #5174), Histone H3 (Cat #4499), Histone H4 (Cat #2935), Acetyl-Histone H3 (Ac-H3, Cat #8173), Acetyl-Histone H4 (Ac-H4, Cat #2594), HLA-DRA (Cat #97971T), CD13 (Cat #32720S) and CIITA (Cat #3793S) were obtained from Cell Signaling Technology (Beverly, MA, United States). Antibody against CD59 (BF0017) was purchased from Affinity Biosciences (Affinity Biosciences, OH, United States). The 2×SYBR Green qPCR Mix (cat #AH0104-B), SPARKscript II RT Plus kit (cat #AG0304-B), Spark ECL Plus A (cat #ED0015-C), Spark ECL Plus B (cat #ED0016-C) and RIPA buffer (SparkJade EA0002) were purchased from SPARKJADE (Shandong, China).

### Cell Lines and Cell Culture

MOLM-13 (M5 subtype of AML expressing MLL-AF9 fusion gene, DSMZ No.: ACC 554), THP-1 (M5 subtype of AML expressing MLL-AF9 fusion gene, DSMZ No.: ACC 16), MV4-11 (AML expressing MLL-AF4 fusion gene, DSMZ No.: ACC 102), and SEM (ALL carrying MLL-AF4 fusion gene, DSMZ No.: ACC 546) cell lines were used. These cell lines were grown in RPMI-1640 medium at 37°C with 5% CO_2_, and the medium contained fetal bovine serum (10%) and streptomycin/penicillin (1%).

### Cell Proliferation Assay

The CCK-8 assay was used to detect the proliferation of MOLM-13, THP-1, MV4-11 and SEM cells. Briefly, cells were cultured in 96-well plates at approximately 5,000 cells/well for 24 h and were treated with I1 or SAHA (0–10 μM). After 72 h, 5 μM CCK-8 was added to each well and placed in a 37°C incubator for 4 h. An Opsys microplate reader (Dynex Technologies, Chantilly, Virginia, United States) was used to measure the light absorbance at 450 nm. The results are expressed as percentage of cell proliferation normalized to DMSO-treated control cells.

### Colony Formation Assay

MOLM-13, THP-1, MV4-11 and SEM cells were incubated with I1 (0–2 μM) in a sterile 24-well flat-bottomed culture plate containing 2.6% methylcellulose medium and 10% FBS for 15 days. A CX43 microscope (Olympus, Shinjuku, Tokyo, Japan) and an Olympus EP50 camera (Olympus, Shinjuku, Tokyo, Japan) were used to count the number of colonies. A colony consisting of approximately 50 cells was counted.

### Cell Cycle Analysis

MOLM-13, MV4-11, THP-1 and SEM cells were incubated with indicated concentration of I1 for 24, 48 or 72 h. After collecting the cells, they were fixed with 70% pre-chilled ethanol and stored at −20°C for at least 24 h. Then cells were washed with PBS, then stained with 50 mg/ml PI and 100 mg/ml RNase A in the dark at room temperature for 30 min. Finally, the Beckman-Coulter DXFLEX flow cytometer (Miami, Florida, United States) was used to detect the percentage of cells in G0/G1, S, and G2/M phases, and the data was analyzed and fitted using ModFit software.

### Cell Apoptosis Analysis

MOLM-13, THP-1, MV4-11 and SEM cells were treated with I1 (0–2 μM) or indicated concentration of SAHA for 72 h. Cells were collected and resuspended in 1× binding buffer then incubated with FITC/Annexin V and PI double labeling in the dark at room temperature for 30 min. Finally, the apoptotic rate was detected by a Beckman-Coulter DXFLEX flow cytometer.

### Analysis of Cell Morphology

MOLM-13, THP-1, MV4-11 and SEM cells were cultured with indicated concentration of I1 for 72 h, and then the cells were collected. Slides were made by cytospin and then air-dried. The cells were stained with Wright-Giemsa, and their morphological characteristics were observed with an optical microscope.

### Cell Surface Antigens Measurement

The MOLM-13, THP-1, MV4-11 and SEM cells were treated with indicated concentration of I1 for 72 h, and the cells were collected and incubated with different antibodies and placed in the dark at room temperature for 30 min, then the expression of differentiation markers was determined by a Beckman-Coulter DXFLEX flow cytometer. Finally, the mean fluorescence intensity (MFI) of antigens of each experiment groups were compared with that of control using the SPSS software.

### mRNA-Sequencing Analysis

mRNA-Sequencing (mRNA-seq) was performed for THP-1 and MOLM-13 cell lines. After 48 h of incubation with indicated concentration of I1, the cells were collected for RNA extraction. Sequencing libraries were prepared and then sequenced. The mRNAs expression levels were determined using FPKM (fragments per kilobase of exon per million fragments mapped). Differential expression analysis was performed using DESeq R packages. The threshold for differential expressions of genes (DEGs) is set as a corrected *p* value of 0.05 and absolute value of log_2_ FC (fold change) ≥ 0.58. The method of geometric test was used to enrich the DEGs from the Gene Ontology and Kyoto Encyclopedia of Genes and Genomes (KEGG) databases for both cell lines. The clusterProfiler package of R software was used to the enrichment analysis of DEGs. The pathways with a FDR value of <0.05 were considered significantly enriched.

### Verification of Expression of Differential Genes by Real-Time PCR

Trizol reagent was used to extract total RNA from MOLM-13 and THP-1 cells treated with indicated concentration of I1 for 24, 48, or 72 h. Reverse transcription of the first cDNA strand was performed using the Primerscript RT kit. The following primers are used to amplify cDNA: forward GAPDH 5′-TGG​GTG​TGA​ACC​ATG​AGA​AGT-3′, reverse GAPDH 5′-TGA​GTC​CTT​CCA​CGA​TAC​CAA-3′; forward CD59 5′-CAG​TGC​TAC​AAC​TGT​CCT​AAC​C-3′, reverse CD59 5′-TGA​GAC​ACG​CAT​CAA​AAT​CAG​AT-3′; forward CD13 5′-GAA​GGA​CAG​CCA​GTA​TGA​GAT​G-3′, reverse CD13 5′-GGA​TAA​GCG​TGA​TGT​TGA​ACT​C-3′; forward HLA-DRA 5′-AGT​CCC​TGT​GCT​AGG​ATT​TTT​CA-3′, reverse HLA-DRA 5′-ACA​TAA​ACT​CGC​CTG​ATT​GGT​C-3′; forward CIITA 5′-CCT​GGA​GCT​TCT​TAA​CAG​CGA-3′, reverse CIITA 5′-TGT​GTC​GGG​TTC​TGA​GTA​GAG-3′.

### Western Blotting Analysis

After treatment of MOLM-13 and THP-1 cells with indicated concentration of I1 for 72 h, the cells were collected and lysed with RIPA buffer containing protease inhibitors. The protein lysate was separated by sodium dodecyl sulfate-polyacrylamide gel (SDS-PAGE) and then transferred to PVDF membrane. After blocking the PVDF membrane with 10% skimmed milk, the membrane was incubated with specific primary antibodies at 4°C overnight, and incubated with goat anti-rabbit or goat anti-mouse immunoglobulin G (lgG) antibody for 1 h at room temperature. Finally, the enhanced chemiluminescence (ECL) reagent detection system was used to visualize the protein expression.

### Statistical Analysis

All experiments were repeated for three times. All data were expressed as mean ± standard deviation (SD). One-way ANOVA was used for the comparison of experiment groups with the control using the SPSS software. *p* < 0.05 was considered statistically significant.

## Results

### I1 Possesses Significant Anti-proliferation Activity Against AML and ALL Cells With MLL Gene Rearrangements

The effect of I1 on cell proliferation compared with SAHA was determined by CCK-8 assay. As shown in Figure 1B, I1 significantly reduced the proliferation of MOLM-13, THP-1, MV4-11 and SEM cells with the IC_50_ value of 1.04, 0.75, 0.58, and 0.87 μM, respectively, that was comparable with that of SAHA 0.26, 0.63, 0.44, and 0.28 μM, respectively. These results indicate that I1 can effectively inhibit the proliferation of AML and ALL cells with MLL gene rearrangements. Moreover, I1 has a similar potency of anti-proliferation as SAHA on these cells.

### I1 Markedly Inhibits Colony Formation of AML and ALL Cells With MLL Gene Rearrangements

**FIGURE 2:**
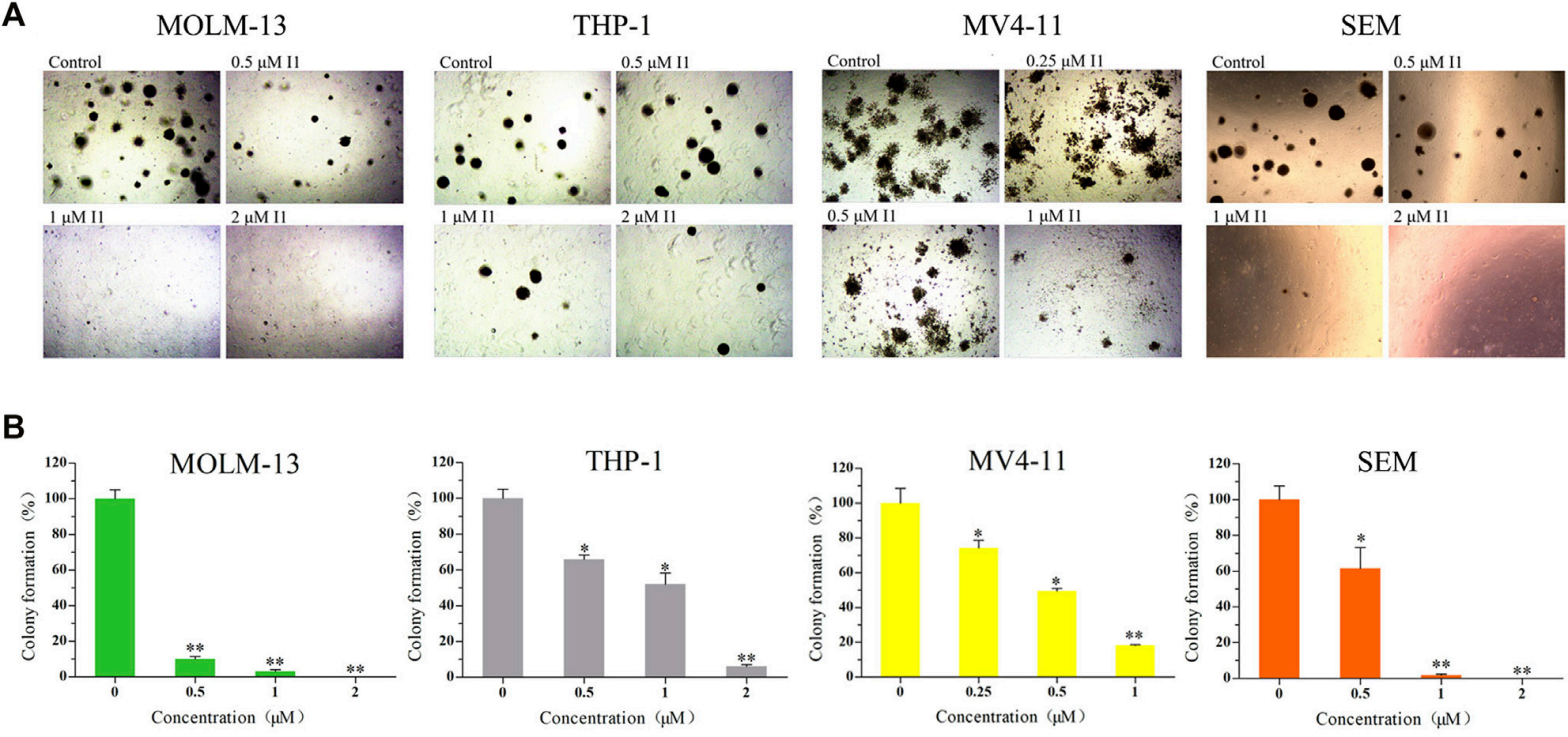
I1 suppressed the colony formation of MOLM-13, THP-1, MV4-11 and SEM cells. **(A)** Cells were treated with I1 at the concentrations of 0.25–2 μM for 15 days then the cell morphology was observed under light microscopy. **(B)** Graph bars exhibited the statistical analysis of the number of colonies (*p < 0.05, **p < 0.01). The colony formation assay was performed three times.

The effect of I1 on the colony forming ability of MOLM-13, THP-1, MV4-11 and SEM cells was explored. As shown in Figures 2A,B, I1 at 0.25–2 µM remarkably inhibited colony formation capacity of MOLM-13, THP-1, MV4-11 and SEM cells in a concentration-dependent manner. In addition, it was found that these cells rarely formed colonies when they were treated with I1 at a concentration of 2 μM. This result indicates that I1 could significantly inhibit the colony-formation capacity of AML and ALL cells with MLL gene rearrangements at a low concentration.

### I1 Induces Cell Cycle Arrest at G0/G1 in AML and ALL Cells With MLL Gene Rearrangements

**FIGURE 3:**
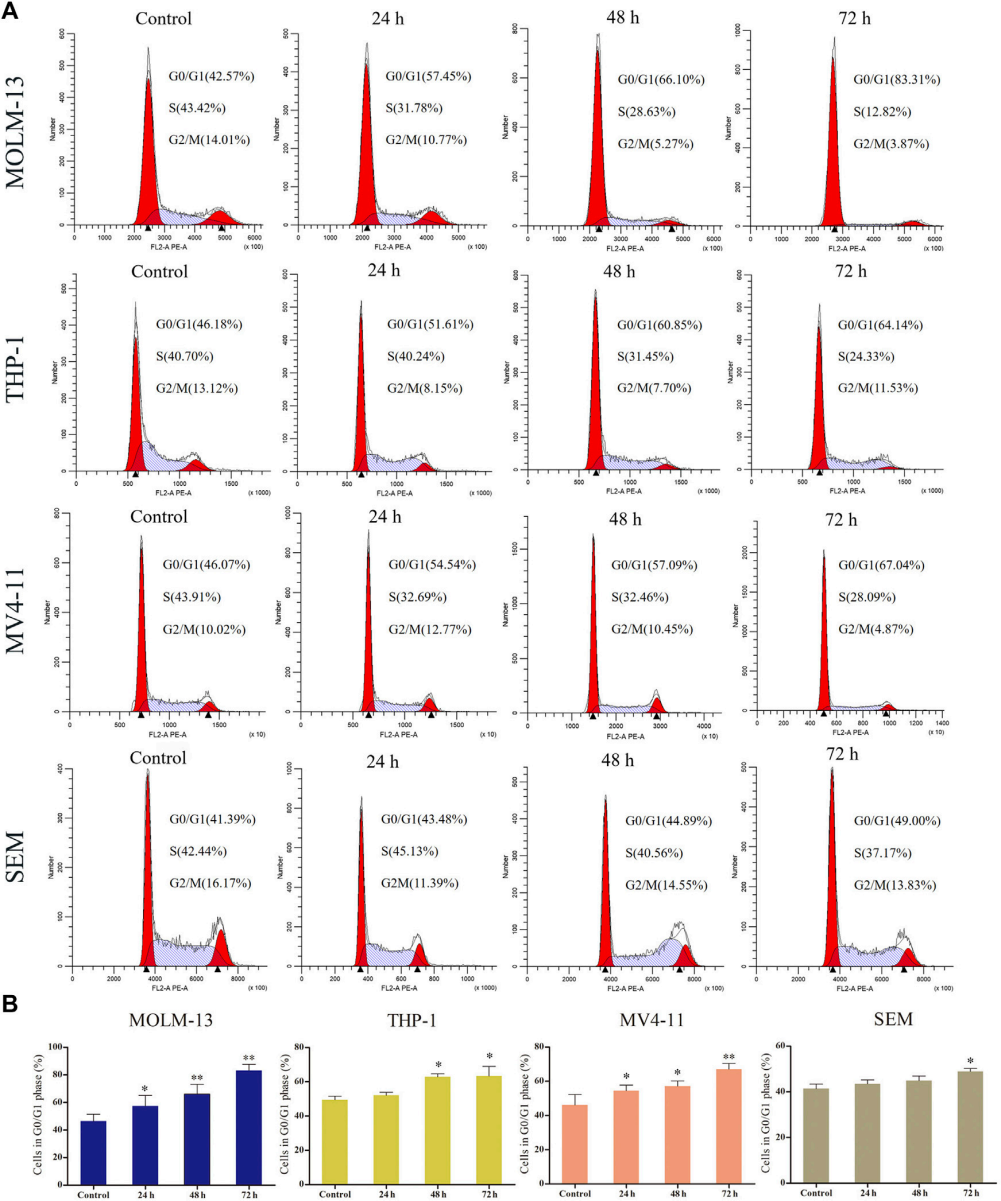
I1 induces cell-cycle exit of MOLM-13, THP-1, MV4-11 and SEM cells. **(A)** MOLM-13, THP-1, MV4-11 and SEM cells were treated with I1 at the concentrations of 1, 0.7, 0.5 or 0.7 μM, respectively for 24, 48 or 72 h, then stained with PI, and detected using flow cytometry. **(B)** Graph bars show the percentage of cells at G0/G1 phase. (*p < 0.05, **p < 0.01). The cell cycle analysis was performed three times.

The effect of I1 on the cell cycle progression of MOLM-13, THP-1, MV4-11 and SEM cells was evaluated. As shown in Figures 3A,B, the percentage of cells in G0/G1 phase increased in these cells with increasing treatment time. This result suggests that I1 inhibits cell proliferation through inducing a G0/G1 cell cycle exit in MLLr -AL cells.

### I1 Induces Minimal Signs of Apoptosis in AML and ALL Cells With MLL Gene Rearrangements

**FIGURE 4:**
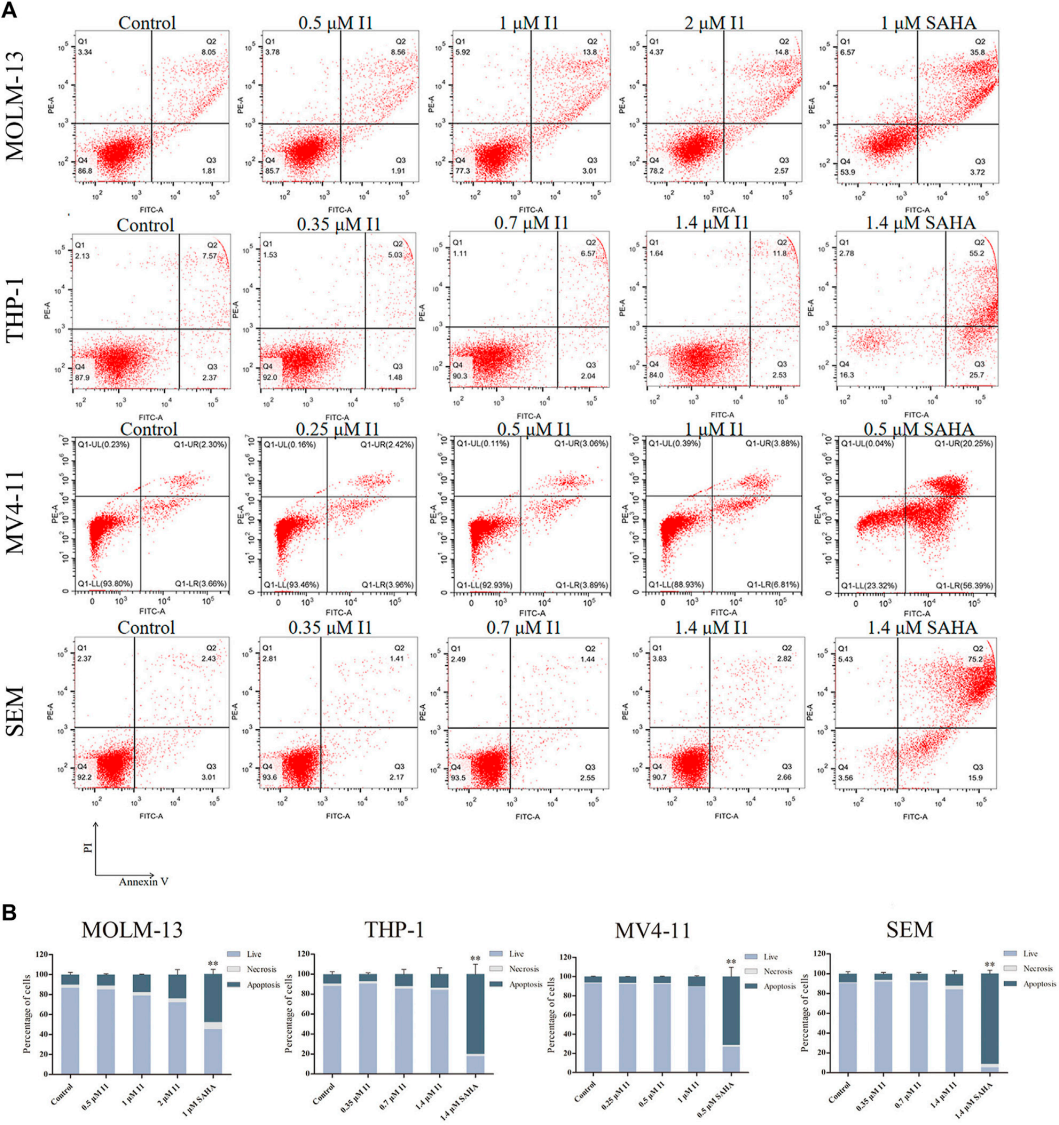
I1 does not induce apoptosis in MOLM-13, THP-1, MV4-11 and SEM cells. **(A)** Cells were treated with I1 or SAHA for 72 h and cell apoptotic rate was determined quantitatively using a flow cytometric apoptosis assay. **(B)** Graph bars show the statistical analysis of apoptosis ratio. MOLM-13 cell was treated with 0.5, 1, 2 μM I1 or 1 μM SAHA. MV4-11 cell was treated with 0.25, 0.5, 1 μM I1 or 0.5 μM SAHA. THP-1 and SEM cells were treated with 0.35, 0.7, 1.4 μM I1 or 1.4 μM SAHA for 72 h (**p < 0.01). The analysis of cell apoptotic rate was performed three times.

In order to determine whether the anti-proliferative effect of I1 on MOLM-13, THP-1, MV4-11 and SEM cells is related to the induction of apoptosis, these cells were treated with indicated concentrations of I1 or SAHA for 72 h. As shown in Figures 4A,B, no significant apoptosis was observed when MOLM-13, THP-1, MV4-11, and SEM cells were incubated with I1 at a concentration of no higher than 2, 1.4, 1 or 1.4 μM, respectively. In contrast, SAHA induced obvious apoptosis in all cell lines at a specific concentration, which may suggest that the mechanism for the anti-proliferation activity of I1 and SAHA is different, particularly in THP-1 and MV4-11 cells. This is consistent with the finding that SAHA induced apoptosis of acute myeloid leukemia cell line HL-60 coupled with at G0/G cell cycle arrest (Wang et al., 2007). This data indicates that the cell cycle arrest was not associated with cell apoptosis in MOLM-13, THP-1, MV4-11 and SEM cells treated with I1 at 1, 0.7, 0.5, 0.7 μM, respectively. These concentrations of I1 is used in the following experiment to explore its effect on cell differentiation. Since SAHA inhibited the proliferation of AML cell line by inducing apoptosis but not cell differentiation, we did not use it as positive control in the following experiments including morphology, the expression of differentiation markers and mRNA-seq.

### I1 Promotes Cell Differentiation in AML and ALL Cells With MLL Gene Rearrangements

**FIGURE 5:**
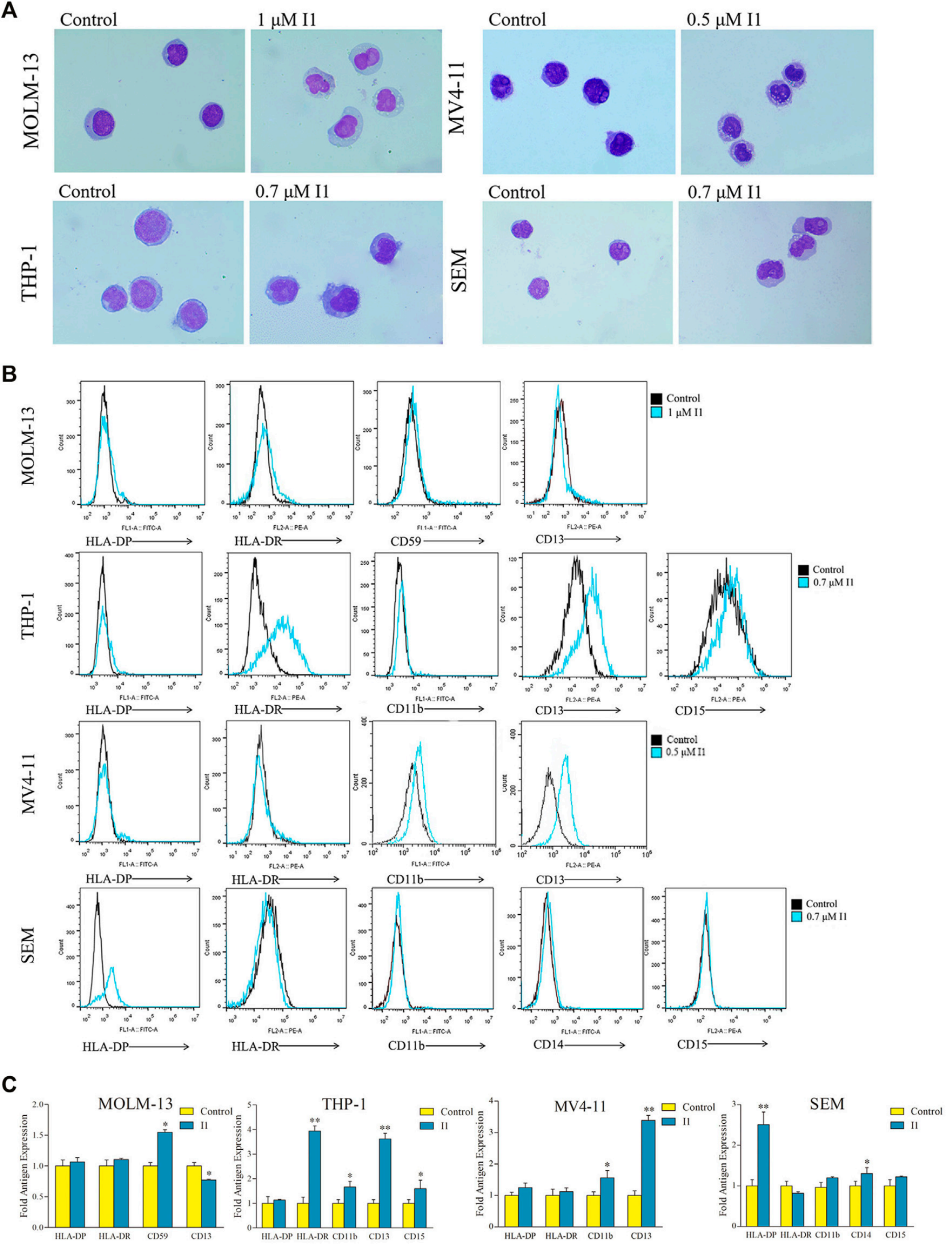
I1 induces differentiation of MOLM-13, THP-1, MV4-11 and SEM cells. **(A)** The morphology of Wright-Giemsa stained cells examined by oil immersion lens (×1,000). **(B)** The expression of different antigens of cells measured by flow cytometry. **(C)** Graph bars show the mean fluorescence intensity (MFI) of antigens. MOLM-13, THP-1, MV4-11 and SEM cells were incubated with 1, 0.7, 0.5 or 0.7 μM I1, repectively for 72 h (*p < 0.05, **p < 0.01). The figures are representative of three independent experiments.

Since I1 did not induce apoptosis of MOLM-13, THP-1, MV4-11 and SEM cells at indicated concentrations, the morphological change and cell surface antigen were analyzed to evaluate the differentiation of these cells treated with I1. The cell phenotype was analyzed by evaluating a set of six differentiation biomarkers (CD11b (a monocyte/granulocyte marker), CD13 (a macrophage/monocyte marker), CD14 (a monocyte/macrophage monocyte/macrophage), CD15 (a monocyte/macrophage marker), CD59 (an erythrocyte marker), HLA-DP and HLA-DR (major histocompatibility complex (MHC) class II genes, immune regulation antigens)). It was found that all the cells showed increased cell size and decreased nuclear/cytoplasmic ratio, indicating that I1 induced cell differentiation with morphological changes (Figure 5A). Moreover, I1 increased the expression of CD59 while decreased the expression of CD13 in MOLM-13 cells. Similarly, I1 increased the expression of CD11b, CD13, CD15, and HLA-DR in THP-1 cells. In addition, the expression of CD11b and CD13 was up-regulated in the MV4-11 cells incubated with I1. Similarly, the expression of CD14 and HLA-DP increased in the SEM cells incubated with I1 (Figures 5B,C). These results indicate that I1 induces differentiation of AML and ALL cells with MLL gene rearrangements.

### I1 Induces Cell Differentiation Through Activating Hematopoietic Cell Lineage Signaling Pathway in MOLM-13 and THP-1 Cells

**FIGURE 6:**
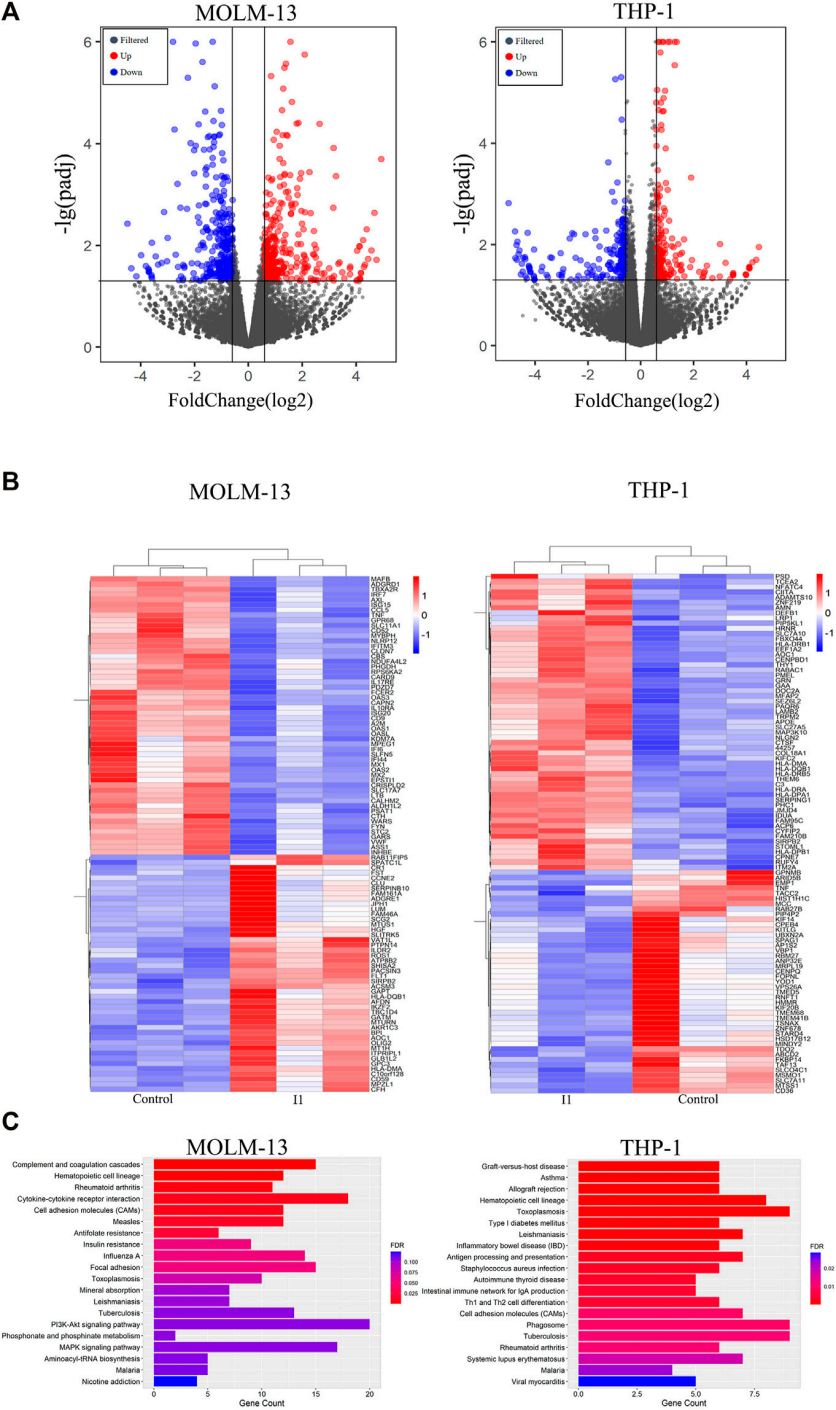
I1 promotes cell differentiation *via* activating hematopoietic cell lineage pathway in MOLM-13 and THP-1 Cells. **(A)** Volcano plots of MOLM-13 and THP-1 cells. **(B)** The heatmap of differentially expressed genes (DEGs). The bars from blue to red denotes the expression levels of DEGs from low to high. (100 genes with *p* < 0.05 and |log 2 FC| > 0.58 based on their *p* value in both cell lines). **(C)** KEGG pathway analyses on all DEGs. MOLM-13 and THP-1 cells were incubated with 1, or 0.7 μM I1, respectively for 48 h. The figures are representative of three independent experiments.

We performed global gene expression analyses using mRNA-seq to explore the molecular mechanism of I1-mediated cell differentiation in MOLM-13 and THP-1 cells. It was found that 352 genes were up-regulated and 329 genes were down-regulated in MOLM-13 cells. Similarly, the expression of 175 genes increased and the expression of 212 genes decreased in THP-1 cells, indicating that I1 has different effects on the expression of genes. The volcano maps of these genes in MOLM-13 and THP-1 are shown in Figure 6A. This result suggested that the effect of I1 on the mRNA expression of all genes is not universal in these cells. As shown in Figure 6B, CD59, HLA-DQB1, HLA-DMA, CR1 and IL7R were significantly up-regulated, while ANPEP (CD13), CD9, FCER2 (CD23) and TNF were markedly down-regulated in MOLM-13 cells incubated with I1. Similarly, HLA-DRA, HLA-DQB1, HLA-DRB5, and CIITA were significantly up-regulated whereas CD36, KITLG and TNF were markedly down-regulated in THP-1 cells treated with I1. Furthermore, the KEGG analysis showed that these differentially expressed genes (CD59, HLA-DQB1, HLA-DMA, CR1, IL7R, CD13, CD9, CD23 and TNF) were enriched in hematopoietic cell lineage signaling pathway in MOLM-13 cells, and HLA-DRA, HLA-DQB1, HLA-DRB5, CD36, KITLG and TNF genes were also enriched in hematopoietic cell lineage signaling pathway in THP-1 cells (Figure 6C).

**FIGURE 7:**
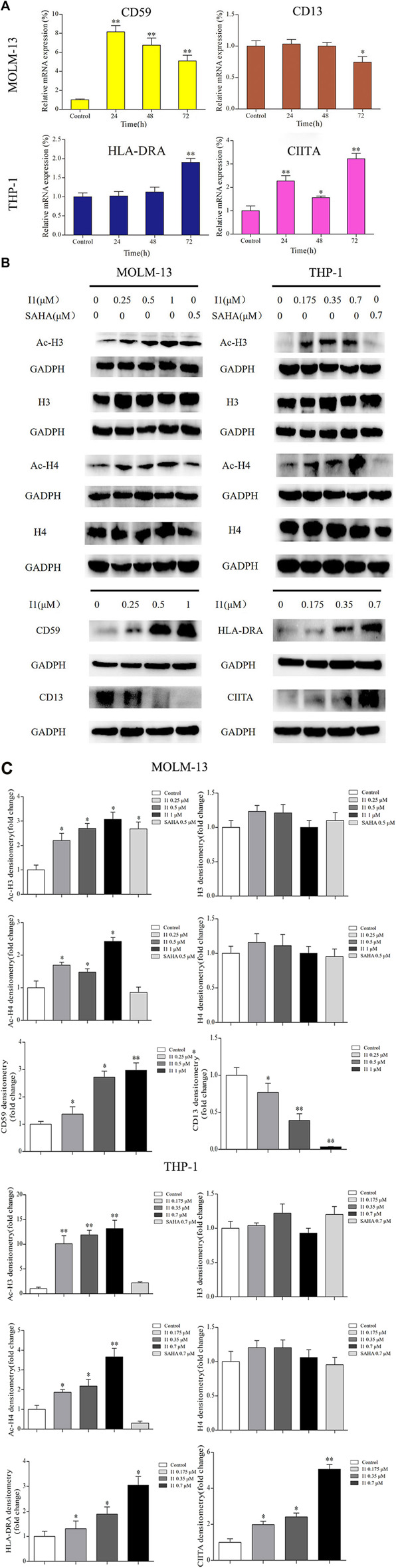
The RT-PCR and Western blotting analysis of cell differentiation related genes in MOLM-13 and THP-1 cell lines. **(A)** The effects of I1 on the mRNA expression of CD59, CD13, HLA-DRA and CIITA evaluated by RT-PCR. MOLM-13 and THP-1 cells were incubated with 1, or 0.7 μM I1 repectively, for 24, 48, or 72 h **(B)** H3, Ac-H3, H4, Ac-H4, CD59, CD13, HLA-DRA and CIITA protein levels measured by Western blotting analysis. **(C)** Graph bars show the protein expression visualized and quantified by AI600 imager. MOLM-13 were incubated with 0.25, 0.5, 1 μM I1, or 0.5 μM SAHA for 72 h. THP-1 cells were incubated with 0.175, 0.35, 0.7 μM I1, or 0.7 μM SAHA for 72 h(*p < 0.05, **p < 0.01).

In addition, some representative differentially expressed genes screened by mRNA-seq were confirmed by RT-PCR and Western blotting in MOLM-13 and THP-1 cells. As shown in Figures 7A–C, I1 treatment significantly changed the expression of CD59 and CD13 mRNA and protein in MOLM-13 cells. In THP-1 cells, the transcription and protein levels of HLA-DRA and CIITA were significantly up-regulated. These results are consistent with the expression identified by mRNA-seq.

Since I1 is a HDAC inhibitor, we explored the effect of I1 on the activity of HDAC inhibition by determining the level of acetylated histone protein H3 and H4 *via* Western blotting analysis. As shown in Figures 7B,C, the acetylated histone H3 and H4 increased in a concentration-dependent manner in MOLM-13 and THP-1 cells treated with I1. Moreover, at the concentration of 1 and 0.7 μM, at which I1 did not induce cell apoptosis but promoted cell differentiation of MOLM-13 and THP-1 cells, respectively, the level of acetylated H3 and H4 is higher in THP-1 cells than that in MOLM-13 cells. In contrast, the same concentration of SAHA did not increase in the acetylated histone H4 in MOLM-13 cells and did not alter the level of acetylated histone protein H3 and H4 in THP-1 cells. This data suggested that the activity of HDAC inhibition of I1 is stronger than that of SAHA in MOLM-13 and THP-1 cells at comparative treatment concentration.

## Discussion

It is well documented that about 10%–20% of patients with acute leukemia carry chromosomal rearrangements involving the MLL gene (Pui et al., 2002; Xu et al., 2020). MLLr-AML mainly occurs in young-to-middle-aged adults whereas MLLr-ALL manifests predominantly in infants younger than 1- year-old. Despite great improvements in the treatment of AML and ALL, literature data suggest that most patients with MLLr-AL have poor clinical outcome (Balgobind et al., 2009; Szczepański et al., 2010). Therefore, there is a pressing need for the development of alternative or complementary therapies against MLLr-AML and MLLr-ALL. Many efforts have been made to develop inhibitors for MLL-MEN1 and MLL-LEDGF interactions, the SET domain of MLL and AF4-MLL, DOT1L and BET proteins (BRD4) (Marschalek, 2015). However, further clinical evaluation is required for these new drugs. Moreover, the impact of these new drugs on both MLL and DOT1L must be very carefully assessed in order to avoid side-effects.

Epigenetic modification regulates gene expression in the absence of alterations in DNA sequences but through nucleosome structural modification (Vidal et al., 2014). Histone modification, a key mechanism of epigenetic regulation, control gene expression by changing the configuration of chromatin and modifying the access of transcription factors to gene promoters (Li et al., 2021). The histone modifications include methylation, acetylation, phosphorylation, ubiquitination, ADP-ribosylation, and sumoylation. Among these, histone acetylation and methylation are most commonly assessed (Manal et al., 2016). Histone deacetylases (HDACs) are chromatin-remodeling enzymes whose enzymatic activity controls the acetylation of histone or non-histone proteins through its influence on chromatin conformation (Johnstone, 2002). Furthermore, histone acetylation affects the transcription and gene replication to activate gene expression. Thus, HDACs play a key epigenetic function as they remove acetyl group from histone or non-histone proteins to regulate gene expression. Therefore, HDACi have been shown to own the activity to induce differentiation or apoptosis and to inhibit cell proliferation in solid or hematopoietic cancers *via* the acetylation of histone or non-histone proteins both *in vitro* and *in vivo* (Göttlicher et al., 2001; Nebbioso et al., 2005). HDACis are generally divided into five groups based on the chemical structure, including hydroxamic acids, cyclic peptides, aminobenzamides, carboxylic acids, and hybrid molecules.

Compared with SAHA (Suberoylanilide hydroxamic acid), I1 is an indole-3-butyric acid bearing HDAC inhibitor with phenyl group in the linker. Similarly, hydroxamic acid group was utilized as the zinc binding group of I1 (Figure 1A). Our previous study demonstrated that I1 exhibited considerable HDAC inhibitory activity compared with SAHA in a study which investigated the enzyme inhibitory potency using HeLa nuclear extract containing a mixture of HDAC isoforms (Chen et al., 2021). This study at the first study showed that I1 had the differentiation-inducing activity in MLLr-AML and MLLr-AL cells. We showed that I1 significantly inhibited the cell proliferation and colony forming ability of MOLM-13, THP-1, MV4-11 and SEM cells by inducing cell differentiation, which is confirmed by the morphological changes and alteration on the expression of the cell surface antigens CD11b, CD13, CD14, CD15, CD59, HLA-DP or HLA-DR. Furthermore, cell differentiation was accompanied by G0/G1 cell cycle exit. Mechanistically, it was revealed that the hematopoietic cell lineage signaling pathway was engaged in the MOLM-13 and THP-1 cells treated with I1. Moreover, it is revealed that I1 showed marked HDAC inhibitory activity through the acetylation of histone H3 and H4 in MOLM-13 and THP-1 cells. In addition, the effect of I1 on the HDAC inhibition activity in THP-1 cells is higher than that in MOLM-13 cells, which is consistent with the IC_50_ values of I1 towards MOLM-13 and THP-1 cells. Taken together, we found that I1 induces cell differentiation and inhibits cell proliferation in MLLr-AML and MLLr-ALL cells.

Genes including CD59, CD13, HLA-DQB1, HLA-DMA, CR1, IL7R, CD9, CD23 and TNF were significantly enriched in hematopoietic cell lineage signaling pathway in MOLM-13 cells. HLA-DRA, HLA-DQB1, HLA-DRB5, CD36, KITLG and TNF were enriched in the signaling pathway in THP-1 cells. It is known that hematopoietic cell lineage signaling pathway, a differentiation-related pathway, denotes the development and differentiation of the hematopoietic cells into various cell types of hematopoietic lineages such as erythrocytes, neutrophils, basophils, eosinophils, macrophages, and myeloid derived dendritic cells (Tenen, 2003; Zhang et al., 2017). Our present study showed that I1 treatment induced cell differentiation with morphological changes, increasing the expression of CD59 and HLA-DR and decreasing the expression of CD13 in MOLM-13 cells. Similarly, I1 up-regulated the expressions of HLA-DR, CD11b, CD13, CD15 in THP-1 cells. I1 treatment also significantly increased the mRNA and protein level of CIITA (a HLA trans-activator) and HLA-DRA in THP-1 cells. This data was in consistent with the finding that up-regulation of CIITA expression enhances the expression of HLA class II antigens (Lazzaro et al., 2001). Therefore, I1-mediated differentiation of MOLM-13 and THP-1 cells might be associated with the hematopoietic cell lineage pathway.

As mentioned above, I1 exhibited significant HDAC inhibitory activity assessed by the acetylation of histone H3 and H4 in MOLM-13 and THP-1 cells. Moreover, growing evidence suggests that HDACi such as valproic acid (VPA) and trichostatin A (TSA), and chromatin-remodeling agents, induced myeloid precursors committed to cell differentiation (Milhem et al., 2004; Bug et al., 2005; De Felice et al., 2005). It reveals that cell fate involved in lineage commitment might be dictated by targeting enzymes with chromatin-remodeling activity such as HDACs (Zardo et al., 2008). Therefore, I1 treatment induces cell differentiation likely originate from the HDAC inhibition activity, as assessed by the acetylation of histone H3 and H4, which may trigger the hematopoietic cell lineage pathway.

In conclusion, our findings show that the HDAC inhibitor I1, as a chromatin-remodeling agent, has a marked anti-proliferative effect on MLLr-AML and MLLr-ALL cells by inducing cell differentiation. Importantly, I1 presented the properties of HDAC inhibition and activated the hematopoietic cell lineage signaling pathway. Moreover, the HDAC inhibition effect of I1 is higher than that of SAHA. I1 could overcome the cell differentiation block of MLLr-AL cells, indicating that I1 could be a potential epigenetic drug worth of further investigation including *in vivo* studies and anti-proliferation activity on primary myeloid leukemia cells and development to surmount differentiation block and be effective in MLLr-AL. Moreover, the induction of cell differentiation would be promising for the treatment of AL.

## Data Availability

The datasets presented in this study can be found in online repositories. The names of the repository/repositories and accession number(s) can be found below: https://www.ncbi.nlm.nih.gov; GSE193965.
